# Prediction of Sensitivity and Efficacy of Clinical Chemotherapy Using Larval Zebrafish Patient-Derived Xenografts of Gastric Cancer

**DOI:** 10.3389/fcell.2021.680491

**Published:** 2021-06-07

**Authors:** Jing Zhai, Jiaqi Wu, Yaohui Wang, Ruoyue Fan, Guiping Xie, Fangfang Wu, Yani He, Sitong Qian, Aimin Tan, Xuequan Yao, Mingfang He, Lizong Shen

**Affiliations:** ^1^Department of Surgical Oncology, Jiangsu Province Hospital of Chinese Medicine, Affiliated Hospital of Nanjing University of Chinese Medicine, Nanjing, China; ^2^Institute of Translational Medicine, College of Biotechnology and Pharmaceutical Engineering, Nanjing Tech University, Nanjing, China; ^3^Department of Pathology, Jiangsu Province Hospital of Chinese Medicine, Affiliated Hospital of Nanjing University of Chinese Medicine, Nanjing, China; ^4^Nanjing Amory Biotech Co. Ltd., Nanjing, China

**Keywords:** gastric cancer, zebrafish patient-derived xenografts, chemotherapy, precision cancer medicine, translational research

## Abstract

**Background:**

Perioperative chemotherapy has been accepted as one of the most common approaches for locally advanced gastric cancer. However, the efficacy of chemotherapy varies among patients, and there is no effective method to predict the chemotherapy efficacy currently. We previously established the first larval zebrafish patient-derived xenografts (zPDXs) of gastric cancer as a platform for the translational research and personalized treatment. The objective of this study was to investigate the feasibility of screening individualized chemotherapeutics using the zPDXs.

**Methods:**

We further optimized this zPDXs platform including administration route, drug dosing, and rhythm to develop a stable and reliable protocol for chemotherapeutics screening. Using the novel platform, we investigated the chemosensitivity of 5-fluorouracil, cisplatin, docetaxel, and doxorubicin for gastric cancer patients.

**Results:**

We showed that the engrafted zebrafish retained the original prominent cell components of the corresponding human tumor tissues, and we successfully obtained the results of chemosensitivity of 5-fluorouracil, cisplatin, docetaxel, and doxorubicin for 28 patients with locally advanced gastric cancer. These patients underwent radical gastrectomy for curative intent and 27 cases received postoperative adjuvant chemotherapy. We revealed that the chemosensitivity obtained from zPDXs was consistent with the clinical responses in these patients (*P* = 0.029). More importantly, the responder drug(s) from zPDXs used or not was the only risk factor for early-stage recurrence in these 27 patients (*P* = 0.003).

**Conclusion:**

Our study with the largest sample size so far suggests that larval zPDXs help to predict the chemotherapeutics response and to achieve precise chemotherapy for gastric cancer.

## Introduction

Gastric cancer remains the fourth most common malignancy and the second leading cause of cancer death worldwide (Bray et al., 2018; Yang et al., 2018). Despite the improvements in screening and diagnosis of gastric cancer, it is often diagnosed at advanced stages and carries a poor prognosis (Coburn et al., 2018). Radical surgical resection is the only curative approach for resectable stomach cancer (Van Cutsem et al., 2016). Perioperative chemotherapy has been accepted to be the most common implemented approach for the locally advanced gastric cancer in addition to radical surgery and target therapy. The purpose of perioperative chemotherapy is to eradicate the locoregional microscopic disease, to prevent or reduce recurrence or metastasis, and eventually to improve the disease-free survival or overall survival of gastric cancer patients (Das, 2017; Petrillo and Smyth, 2020; Xiang et al., 2020).

However, the efficacy of perioperative chemotherapy varies greatly from individual to individual, and the benefits from chemotherapy are not consistent (van den Ende et al., 2019). Although several molecular markers, such as mismatch repair deficiency (MMRD) and microsatellite instability (MSI), have been shown to be associated with a prognosis in patients with resectable gastroesophageal cancer (Smyth et al., 2017), there is no effective method to predict the efficacy of perioperative chemotherapy for advanced gastric cancer currently (Qin et al., 2020). Chemoresistance and tumor recurrence remain the major bottlenecks for curing gastric cancer (Mokadem et al., 2019). Current chemotherapy regimens are developed and approved based on the average efficacy and acceptable safety (Fior et al., 2017), and the choice of chemotherapy regimen is mainly based on Lauren classification, WHO classification and pTNM staging of gastric cancer (Zurlo et al., 2020). With the exception of administration of molecular targeted drugs for patients with HER-2 positive gastric cancer (Shitara et al., 2020), the majority of gastric cancer patients receive “one-size-fits-all” chemotherapy following various guidelines, which inevitably leads to huge differences and uncertainties in treatment responses in the individual patients (Petrelli et al., 2019; Tokunaga et al., 2020). The overall efficacy rate of chemotherapy for advanced gastric cancer hovers at 30 to 54% (Smyth et al., 2020), and patients go through rounds of trial-and-error approaches to find the best regimen. It remains challenging to find the individualized effective chemotherapeutics for certain patients among various chemotherapy regimens to improve the treatment response, which remains a challenge for precision medicine in gastric cancer (Bonelli et al., 2019).

*Ex vivo* models such as patient-derived xenografts (PDXs), have been developed for patient-specific drug screening (Gao et al., 2015; Yoshida, 2020). PDXs generally maintain the characteristics of original tumor microenvironment, and retain the heredity background and gene mutation profiles of the tumors (Invrea et al., 2020). PDXs are thought to predict drug responses in patients and thus reflecting the uniqueness of each patient with higher clinical relevance (Barriuso et al., 2015; Invrea et al., 2020). However, mouse PDXs (mPDXs) present significant disadvantages including large numbers of cells required (approximately 10^6^ cells per mouse), expensiveness, lengthy (months) process that introduces genetic and epigenetic changes to the tumor, and a lack of easily accessible real-time monitoring of cells within the mouse, rendering this model less applicable for clinical practice (Xiao et al., 2020). Most recently, the zebrafish PDXs (zPDXs) have been demonstrated to be an ideal tool for personalized medicine (Brown et al., 2017; Fazio et al., 2020). Imaging of the small, transparent fry is unparalleled among vertebrate organisms. The unique advantages, including the speed (5–7 days) and small patient tissue requirements (100–200 cells per fish) of zPDXs, enable patient-specific real-time chemosensitivity analyses (Fazio and Zon, 2017). Fior et al. (2017) demonstrated preliminarily that the response to chemotherapy and biological therapies in the zPDXs of colorectal cancer had 80% clinical relevance with that in the patients. It has been shown that relative sensitivities obtained in zebrafish are maintained in the rodent model (Fior et al., 2017; Yan et al., 2019).

We previously established the first gastric cancer xenografts in living larval zebrafish, and our preliminary study approved that the zPDX model may serve as a promising platform for the translational research and personalized treatment for gastric cancer (Wu et al., 2017). To investigate the feasibility of the zPDXs to screen individualized chemotherapeutics for certain gastric cancer patients, we optimized the platform including administration route, drug dosing, and rhythm to develop a stable and reliable protocol, and further validated that the zPDXs retained the main clinicopathological characteristics of human gastric cancer. We compared the chemosensitivity to 5-fluorouracil (5-FU), cisplatin (CDDP), docetaxel (DXT), and doxorubicin (Dox) in zPDX models with clinical responses for 28 patients with locally advanced gastric cancer. These patients underwent radical gastrectomy for curative intent and 27 cases of them received postoperative adjuvant chemotherapy. We found that the chemosensitivity obtained from zPDXs was consistent with the clinical responses in these patients. More importantly, the responder drug(s) from zPDXs used or not was the only risk factor for early stage recurrence in these 27 patients. Our study with the largest sample size so far suggests that the larval zPDX model is a useful and effective platform to predict the chemotherapeutics response and to achieve precise chemotherapy for gastric cancer.

## Materials and Methods

### Zebrafish Care and Handling

Transgenic zebrafish *Tg(fli-1:EGFP)* expressing enhanced green fluorescent protein (eGFP) in the endothelial cells were obtained from the Model Animal Research Center of Nanjing University. These zebrafish were cared and handled according to our previous report (Wu et al., 2017). Embryos were collected and placed at 28.5°C in Petri dishes containing embryo medium (0.2 g/L of Instant Ocean^®^ Salt in distilled water). The age of the embryos is indicated as hours post fertilization (hpf) or days post fertilization (dpf). The zebrafish studies were approved by the Institutional Animal Care and Use Committee (IACUC) of Nanjing University of Chinese Medicine.

### Cell Line Culture and Primary Tissue Dissociation

The human gastric adenocarcinoma cell lines AGS, SGC7901, and MGC803 (ATCC, United States) were cultured in RPMI1640 supplemented with 10% FBS and 100 U/ml penicillin and streptomycin.

Fifty-six human locally advanced gastric cancer tissue samples, from June 2018 to October 2019, were obtained from the Department of Surgical Oncology of Jiangsu Province Hospital of Chinese Medicine/Affiliated Hospital of Nanjing University of Chinese Medicine. All these patients underwent radical gastrectomy for curative intent, and most of them received postoperative adjuvant chemotherapy. All the sample studies were performed following written consent according to an established protocol approved by the Institutional Review Board of Nanjing University of Chinese Medicine. This study was also in accordance with the Declaration of Helsinki. All enrolled patients did not receive preoperative chemotherapy or radiotherapy. The tissue samples were transferred directly into the pre-chilled tissue storage solution (Miltenyi, BergischGladbach, Germany) after resection. Primary single cells from the tissue samples were isolated using the tumor dissociation kit (Miltenyi, BergischGladbach, Germany) following the manufacturer’s instructions.

### *In vitro* Cell Viability Assay

Cell viability was measured using a cell counting kit-8 (CCK-8, Dojindo, Japan) according to the manufacturer’s instructions.

### Cell Labeling, Xenograft, and Enumeration Procedure

Human cell lines and primary gastric cancer cells were fluorescently labeled with CM-DiI (Invitrogen, Life Technologies, Carlsbad, CA, United States) according to the manufacturer’s instructions. Labeled cells were washed in PBS twice, resuspended in RPMI1640 supplemented with 10% FBS at 2 × 10^7^ cells/ml. Cell viability was assessed by trypan blue staining before the injection. Cell viability was higher than 95% for cell lines and 70% for primary cells. Xenograft and enumeration procedure were performed with reference to our previous report (Wu et al., 2017).

### Drug Administration by Soaking

For drug delivery by soaking, drug exposure by addition to the larval water, xenografted zebrafish embryos at 72 hpf were transferred randomly to 24-well plates, with 10 embryos per well with 0.5 ml of embryo medium containing various concentrations of drugs for a treatment period of 48 h. 5-FU (Sigma-Aldrich, St. Louis, MO, United States), CDDP (Selleck, Houston, TX, United States), DXT (Meryer, Shanghai, China), and Dox (Meryer, Shanghai, China) were used. 5-FU and CDDP were dissolved in embryo medium, while DXT and Dox were dissolved in embryo medium containing 0.1% DMSO. Treatment experiments were carried out at a constant temperature of 32°C in the dark.

### zPDXs Monitoring and Imaging

Tumor cell growth *in vivo* was monitored using an inverted fluorescence microscope (IX71, Olympus, Japan). The cell number at 0 day post treatment (dpt) [1 day post injection (dpi)] was set as the baseline and was normalized to 1, and the cell proliferation was determined by folds on 3 dpt with reference to the cells on 0 dpt.

fold⁢change=tumor⁢cell⁢number⁢in⁢each⁢embryo⁢at⁢ 3⁢dpttumor⁢cell⁢number⁢in⁢each⁢embryo⁢at⁢ 0⁢dpt

### LC-MS/MS Analysis for 5-FU Concentration

Liquid chromatography–mass spectrometry (LC–MS/MS) analysis for 5-FU concentration was performed according to the report of Jones et al. (2012).

### L Immunohistochemistry

L Immunohistochemistry (IHC) assays were conducted according to the standard protocols. A mouse monoclonal anti-carcinoembryonic antigen (CEA) antibody (Dako, Glostrup, Denmark), a mouse monoclonal anti-carbohydrate antigen 199 (CA199) antibody (ZSGB-BIO, Beijing, CN, United States), and a rabbit monoclonal anti-hyaluronan and proteoglycan link protein 1 (HAPLN1) antibody (Abcam, Cambridge, United Kingdom) were used.

### L Statistical Analyses

All statistical analyses were expressed as mean ± SEM using GraphPad Prism 5.0. The decrease/increase in fold of change was analyzed using one-way ANOVA followed by Dunnett multiple comparison test. Significance was considered when *P*-values were less than 0.05. All experiments were done in triplicate and independent experiment was repeated at least three times.

## Results

### L Optimization of the Larval Zebrafish Platform for Preclinical Chemosensitivity Test of Gastric Cancer

**FIGURE 1:**
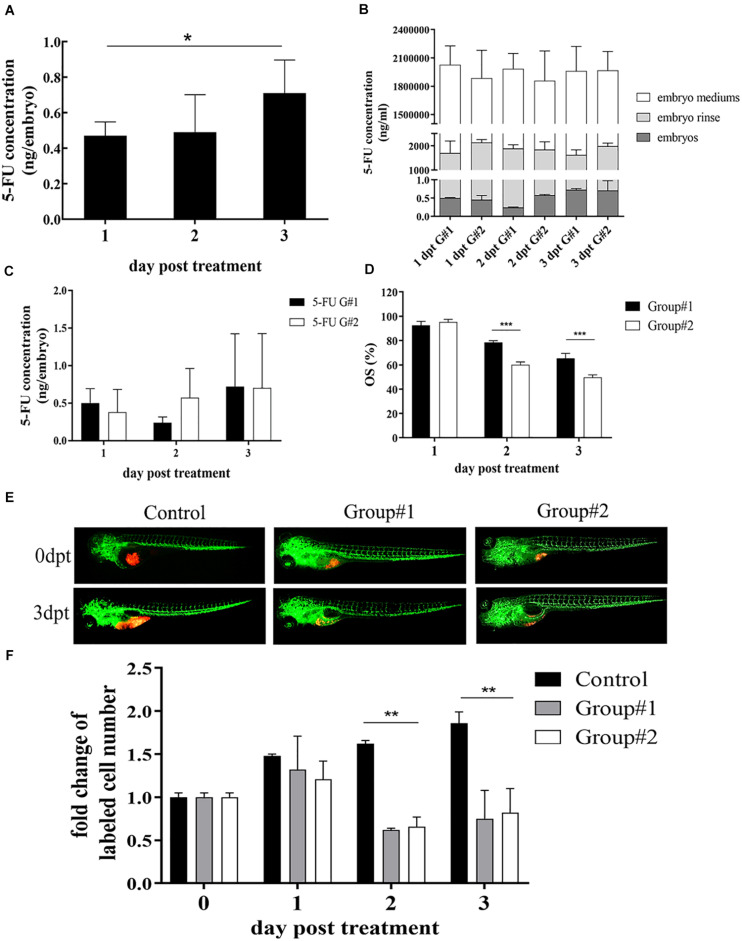
Optimization of 5-FU dosing in zebrafish. **(A)** The internal concentration of 5-FU in embryos engrafted with SGC7901 cells was increased significantly after treatment with 5-FU (5 mM) in embryo medium, and the average internal concentration of 5-FU was 0.47, 0.49, and 0.71 ng per embryo on 1, 2, and 3 dpt, respectively (*P < 0.05). **(B)** Treatment for the embryos without xenograft showed that the internal concentration of 5-FU in embryos increased remarkably in both groups (*P* < 0.05), but the internal concentration was lower than that in embryo mediums and in the embryo rinse, indicating 5-FU enriched in embryos was a tiny fraction of administration [G#1 (group #1), daily refreshment of drug-containing medium; G#2 (group #2), continuous drug-containing medium without refreshment]. **(C)** zCDX study with SGC7901 cells showed that the internal concentration of 5-FU did not show difference between these two groups on each dpt (*P* > 0.05). However, the overall survival (OS) of embryos increased in group #1 (***P < 0.0001) **(D)**. **(E, F)** These two administrations of 5-FU inhibited the tumor cell proliferation in the zCDX with SGC7901 cells (dyed with red fluorescence using DiL) significantly (**P < 0.01), and there was no difference in cell growth inhibition between group #1 and group #2 (*P* > 0.05).

We have previously established zPDXs of gastric cancer (Wu et al., 2017). To screen the preclinical chemosensitivity of drugs for gastric cancer patients, we explored to establish a stable and reliable protocol of drug administration in zebrafish cell-derived xenograft (zCDX) model using human gastric cancer cell lines SGC7901, MGC803, and AGS. In addition, we assessed the chemosensitivity of four categories of chemotherapeutic drugs mainly used in clinical practice for human gastric cancer patients, including 5-FU, CDDP, DXT, and Dox. There are usually two routes of drug delivery in larval zebrafish models, submersion in drug dissolved in water (soaking) and microinjection into the yolk sac. According to the database^1^, the *LogP* of 5-FU, CDDP, DXT, and Dox is −0.89, −2.19, 2.40, and 1.27, respectively. Thus, 5-FU and CDDP are water-soluble and the others are liposoluble. Our previous study have showed that administration of 5-FU *via* microinjection at the maximum tolerated dose (MTD) of 65 ng/embryo inhibited the cell proliferation in zCDXs with AGS or SGC7901 cells, and SGC7901 cells showed more sensitivity than AGS cells to 5-FU as that in *in vitro* assay. However, microinjection, especially repeated microinjection, inevitably causes damages to zebrafish embryos, which may influence the drug assays. Thus, we probed whether 5-FU could be administrated by submersion. AGS or SGC7901 cells were xenografted into zebrafish embryos at 48 hpf, respectively. At 72 hpf, 50–5,000 μM of 5-FU were administrated to these embryos by soaking, and none of the concentrations caused embryo death and induced any adverse effect on the embryo development on 6 dpt (data not shown). The 3 dpf embryos were moved to fresh embryo medium containing 5 mM 5-FU, and the fresh medium was replaced daily in the next 3 days. The embryos, embryo mediums containing drug, and the embryo rinse were collected daily to assay the concentrations of 5-FU by LC-MS/MS. As shown in Figure 1A, the internal concentration of 5-FU in embryos engrafted with SGC7901 cells was increased daily after treatment with 5-FU (*P* < 0.05), and the average internal concentrations of 5-FU were 0.47, 0.49, and 0.71 ng per embryo at 1, 2, and 3 dpt, respectively.

We further investigated the effects of administration rhythm on 5-FU enrichment in the embryos and its therapeutic efficacy. There were two groups, group #1 (daily refreshment of drug-containing medium) and group #2 (continuous drug-containing medium without refreshment). First, treatment for the embryos without xenograft showed that the internal concentration of 5-FU in embryos increased remarkably in both groups (*P* < 0.05, Figure 1B). However, the internal concentration was lower than that in embryo mediums and in the embryo rinse, indicating that 5-FU enriched in embryos was a tiny fraction of administration, which was consistent with our previous reports (Jiang et al., 2016; Li et al., 2017). Next, zCDX study with SGC7901 cells showed that the internal concentration of 5-FU did not show difference between these two groups on each dpt (*P* > 0.05, Figure 1C). However, the overall survival (OS) of embryos increased in group #1 (*P* < 0.0001, Figure 1D), and the embryos without xenograft did not show this difference (data not shown). We further investigated the effects of administration rhythm on 5-FU sensitivity test and found that these two administrations significantly inhibited the tumor cell proliferation in the zCDX with SGC7901 cells significantly (*P* < 0.01), and there was no difference in cell growth inhibition between these two groups (*P* > 0.05) (Figures 1E,F).

**FIGURE 2:**
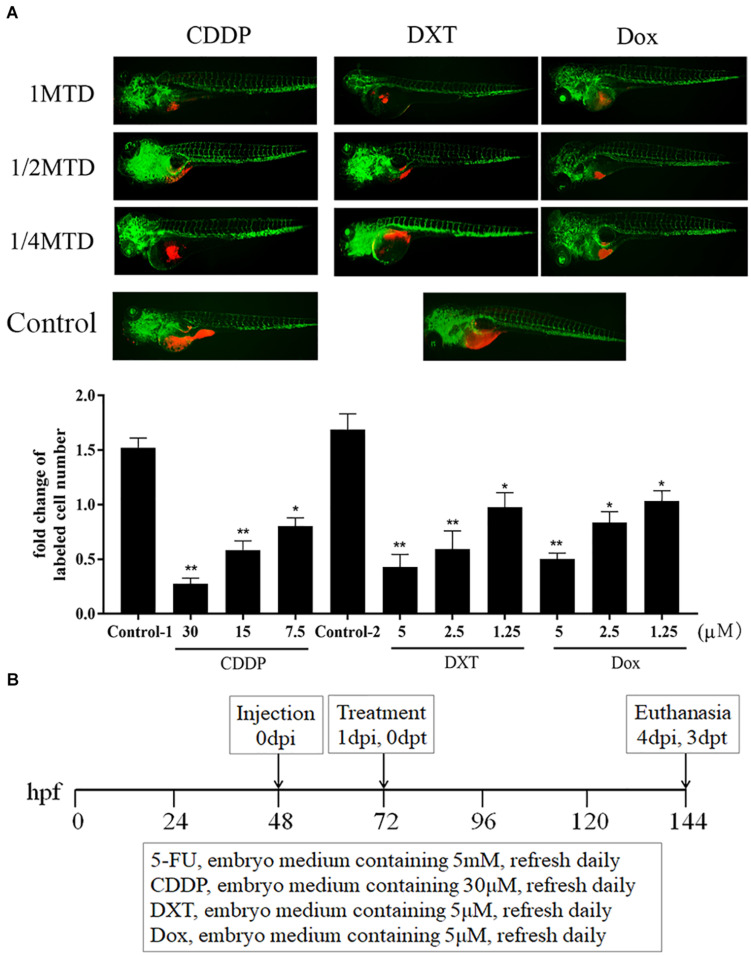
CDDP, DXT, and Dox dosing in zebrafish. **(A)** zCDXs engrafted SGC7901 cells (dyed with red fluorescence using DiL) were treated with CDDP, DXT, or Dox at their 1/4, 1/2, and 1 MTD, and they inhibited cell proliferation in a dose-dependent manner (control-1, embryo medium for CDDP assay; control-2, embryo medium containing 0.1% DMSO for DXT or Dox assay) (*P < 0.05, **P < 0.01 vs. control-1 or control-2). **(B)** A protocol for preclinical chemosensitivity test in the zebrafish larvae cancer model was developed.

Similarly, the MTD of CDDP was determined to be 30 μM for zebrafish embryos. For the liposoluble drugs, we first submersed zebrafish embryos without xenograft in fresh embryo media containing 0.1% DMSO and drugs to evaluate the MTD of each drug (de Koning et al., 2015), and the MTDs of DXT and Dox were 5 μM. These drugs at their MTD were not lethal and did not cause any adverse effect on the embryo development at 3 dpt (data not shown). Then, we treated zCDXs engrafted AGS or MGC803 cells with each drug at its 1/4, 1/2, and 1 MTD. As shown in Figure 2A, CDDP, DXT, or Dox inhibited cell proliferation in a dose-dependent manner, and they, at the dose of MTD, could inhibit cell proliferation significantly (*P* < 0.01). Thus, the zPDXs were dosed at 1 MTD of each drug in the subsequent preclinical study. Based on these results, we developed a protocol for preclinical chemosensitivity test in the zebrafish larvae cancer model as illustrated in Figure 2B.

### zPDX Model of Gastric Cancer Retains the Original Prominent Cell Components of Human Gastric Cancer

**FIGURE 3:**
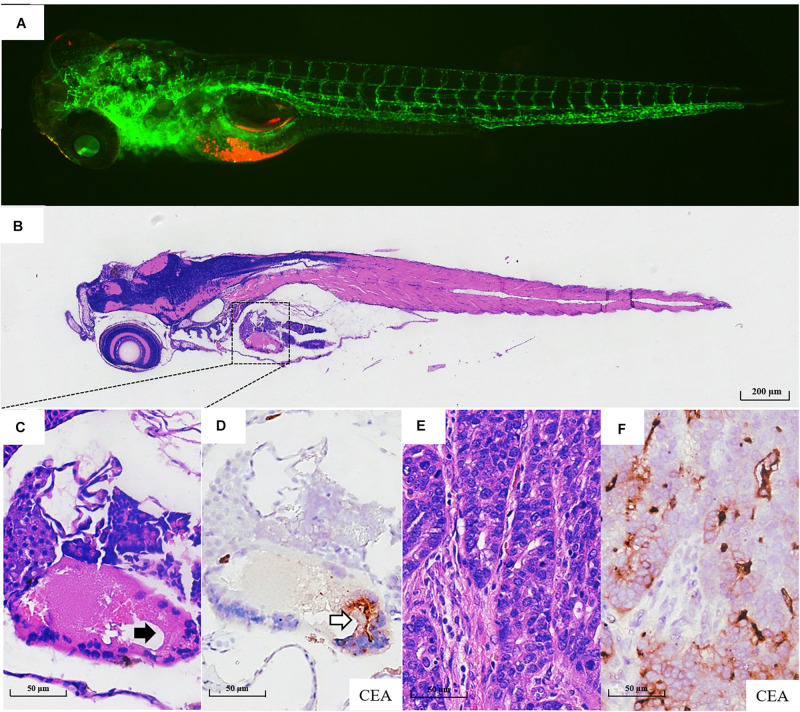
Pathological assays of the engrafted zebrafish for patient #43. **(A)** The confocal image and **(B)** the H & E image of the engrafted zebrafish (4 dpi). **(C)** The enlarged yolk sac showing the tumor xenograft (black arrow indicating engrafted tumor cells), which did not develop to the adenoid structure as that in the patients **(E)**. **(D)** CEA expression in tumor xenograft (blank arrow). **(E)** The H & E image of the primary tumor. **(F)** CEA expression in primary tumor tissue.

**FIGURE 4:**
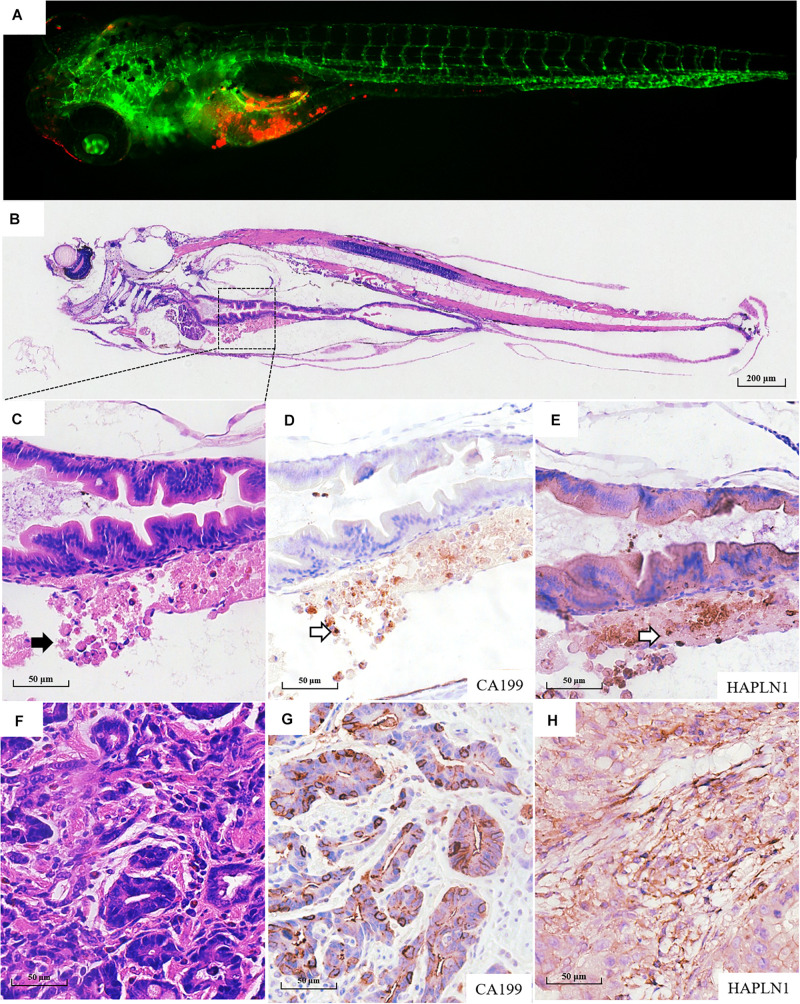
Pathological assays of the engrafted zebrafish for patient #55. **(A)** The confocal image and **(B)** the H & E image of the engrafted zebrafish (4 dpi). **(C)** The enlarged yolk sac showing the tumor xenograft (black arrow indicating engrafted tumor cells), which did not develop to the adenoid structure as that in the patients **(F)**. **(D)** CA199 expression in tumor xenograft (blank arrow) and **(G)** CA199 expression in primary tumor tissue. **(E)** HAPLN1 expression in tumor xenograft (blank arrow) and **(H)** HAPLN1 expression in primary tumor tissue.

Fifty-six patients with locally advanced gastric cancer were enrolled in this study, and the detailed clinicopathological information is listed in Supplementary Table 1. We further validated whether the zPDXs retain the clinicopathological characteristics of human gastric cancer. We investigated tumor cell development and progression in the zPDX models of six gastric cancer patients using morphologic analysis with histological hematoxylin and eosin (H&E) staining. We also assayed for the main cell components of tumor microenvironment with IHC staining in these zPDXs, including CEA or CA199 for cancer cells and HAPLN1 for stromal cells (Figures 3A–F, Figures 4A–F). Unexpectedly, the engrafted zebrafish did not develop histologically similar tumors to those in patients (Figures 3C, 4C). However, the expression statuses of CEA (Figure 3D) and CA199 (Figure 4D) were consistent with that in the corresponding human cancer tissues (Figures 3F, 4G). Importantly, HAPLN1, mainly produced by cancer-associated fibroblasts (CAFs) (Figure 4H), was also detected in the tumor tissues in the zPDX models (Figure 4E). The detailed status of these markers in patient tumor tissues and the corresponding zPDXs are shown in Supplementary Table 2. These findings indicated that the zPDXs conserve the original prominent cell components of human gastric cancer, although some clinicopathological characteristics of human gastric cancer may not be mimicked in the zPDX models.

### Preclinical Chemosensitivity Test in zPDXs of Gastric Cancer Is Consistent With Patient Chemotherapy Response

We next performed preclinical chemosensitivity study for gastric cancer patients in the zPDX models. As shown in Supplementary Figure 1, the gastric cancer tissue samples were collected and the primary single-cell suspensions were prepared. Each embryo was engrafted about 200 cells. The zPDX procedure for human gastric cancer tissues was technique-challenging. Based on our experience, if the engrafted zebrafish embryos were vigorous (the mortality less than 10%) and their numbers were sufficient for evaluating drug sensitivity (at least more than 50 embryos per drug), the zPDX model was considered to be established successfully. The zPDX models were successfully established and chemosensitivity tests were achieved in 28 of 56 enrolled patients (Table 1 and Supplementary Table 3). The main technical problems included sample contamination, unskilled technician, needle blocking during microinjection, and insufficient cell viability (less than 10^6^ cells after primary single cell preparation). After continuous technique improvement, the success rate of zPDX establishment was increased and stabilized at 80%.

**TABLE 1:** The detailed results of successfully established zPDX of gastric cancer patients.

Patient no.	Tumor recurrence^1^			Postoperative adjuvant chemotherapy^2^		Follow-up (m)	zPDX^3^
	Status	Time (m)	Evidence	Regimen	Regimen changing		Chemosensitivity results
#1	NO			NO	NO	20	All resistance
#6	NO			Capecitabine	NO	18	CDDP***, 5-FU**, Dox*, DXT^*r*^
#9	NO			CAPEOX	NO	17	5-FU*, CDDP*, DXT*, Dox^*r*^
#11	NO			FOLFORI	NO	15	DXT***, 5-FU*, CDDP^*r*^, Dox^*r*^
#15	NO			CAPEOX	NO	15	5-FU***, DXT***, CDDP^*r*^, Dox^*r*^
#16	NO			Capecitabine	NO	14	5-FU***, DXT***, CDDP^*r*^, Dox^*r*^
#20	YES	6	AFP↑ (16.40)	TP	Capecitabine	14	5-FU*, DXT^*r*^, CDDP^*r*^, Dox^*r*^
#23	NO			Capecitabine	NO	14	All resistance
#25	NO			FOLFORI	NO	14	5-FU*, DXT^*r*^, CDDP^*r*^, Dox^*r*^
#27	YES	6	CEA↑ (5.40)	FOLFORI	FOLFOX	14	CDDP***, 5-FU***, DXT***, Dox***
#29	NO			Capecitabine	NO	13	5-FU***, CDDP***, Dox***, DTX***
#32	NO			FLOT	NO	13	DXT***, 5-FU***, CDDP^*r*^, Dox^*r*^
#34	NO			Capecitabine	NO	12	5-FU*, Dox^*r*^, CDDP^*r*^, DXT^*r*^
#35	YES	12	CEA↑ (6.25)	FLOT	TP	12	5-FU*, DXT^*r*^, Dox^*r*^, CDDP^*r*^
#36	NO			FOLFORI	NO	12	DXT*, CDDP*, Dox^*r*^, 5-FU^*r*^
#38	NO			DCF	NO	12	5-FU*, DXT^*r*^, Dox^*r*^, CDDP^*r*^
#39	NO			FLOT	NO	11	5-FU*, DTX*, CDDP*, Dox^*r*^
#41	NO			FOLFORI	NO	11	5-FU*, DXT*, CDDP^*r*^, Dox^*r*^
#43	YES	7	CT (lymph nodes)	FLOT	NO	11	All resistance
#45	NO			FOLFOX	NO	11	CDDP*, DXT*, Dox*, 5-FU^*r*^
#47	YES	7	CEA↑ (10.64) Die at 9 m.	Capecitabine	NO	10	All resistance
#48	NO			FOLFOX	NO	10	5-FU*, DXT^*r*^, Dox^*r*^, CDDP^*r*^
#50	NO			FLOT	NO	10	5-FU**, Dox^*r*^, CDDP^*r*^, DXT^*r*^
#52	NO			TP	NO	10	DXT*, CDDP^*r*^, 5-FU^*r*^, Dox^*r*^
#53	NO			Capecitabine	NO	8	5-FU*, CDDP*, Dox^*r*^, DXT^*r*^
#54	NO			Capecitabine	NO	8	CDDP***, DXT***, 5-FU***, Dox^*r*^
#55	YES	5	CT (lymph nodes)	FOLFOX	FLOT	8	All resistance
#56	NO			Capecitabine	NO	8	DXT**, 5-FU**, CDDP^*r*^, Dox^*r*^

^1^Tumor recurrence was monitored continuously by measuring serum tumor markers or image study. “YES” means recurrence, and we showed the recurrence time (months after surgery) and the evidences. ^2^Most patients have undergone postoperative adjuvant chemotherapy, and some patients’ regimens were changed due to recurrence or other reasons. (“NO” means the patient did not receive adjuvant chemotherapy or the patient’s regimen did not change during therapy.) ^3^In this study, the zPDX models were established in 28 cases successfully, and the chemosensitivity of 5-FU, CDDP, DXT, and Dox was obtained (“*–***” means the treatment inhibited cell proliferation significantly, *P < 0.05, **P < 0.01, and ***P < 0.001. “r” means resistance to the drug. “All resistance” means resistance to these four drugs).

We intended to perform the chemosensitivity test in zPDXs to evaluate the responses of patient tumors to certain drugs, rather than to determine the doses of the drugs. If the drug induced significant proliferation inhibition (*P* < 0.05), it was considered that the patient’s tumor has a good response to the drug. As shown in Supplementary Figure 2, treatment of 5-FU, CDDP, DXT, or Dox exerted the inhibition of tumor proliferation in zPDXs of different patients, indicating that the protocol established in the zCDX study was applicable for preclinical zPDX tests.

**FIGURE 5:**
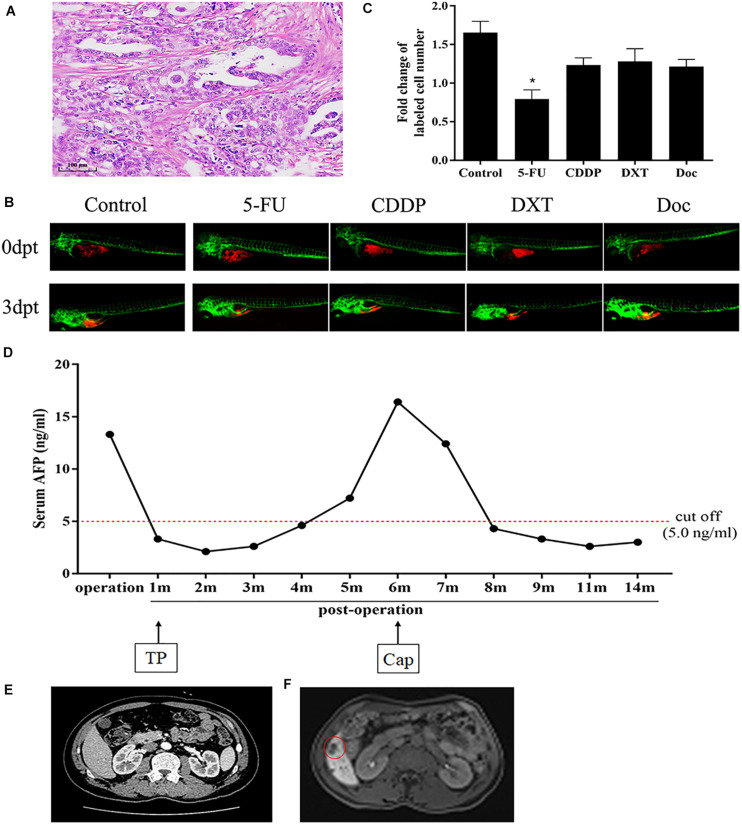
Chemosensitive profiling in zPDXs of patient #20 and its clinical relevance. **(A)** Patient #20 was diagnosed as diffuse-type gastric adenocarcinoma. **(B,C)** His zPDXs study showed that 5-FU was the only sensitive drug (*P < 0.05). **(D)** The patient had elevated preoperative serum AFP level (13.30 ng/ml), and his serum AFP level went down to normal range after radical surgery. He initially received postoperative TP (oxaliplatin/paclitaxel) therapy, but his serum AFP increased to 16.4 ng/ml at postoperative 6 months. **(E)** He received CT scan at postoperative 1 month as baseline and no abnormality was found. **(F)** However, his MR monitoring showed one metastatic foci (red ring) in the right lobe of his liver at postoperative 6 months. Thus, he was considered to relapse. The regimen was adjusted to capecitabine (Cap, a prodrug of 5-FU). Two cycles later, his serum AFP level returned to the normal range, and stayed for more than 5 months.

The detailed results of chemosensitivity assays in zPDXs are listed in Table 1, and the susceptibility rates of 5-FU, CDDP, DXT, and Dox were 57.14% (16/28), 25.00% (7/28), 42.86% (12/28), and 14.29% (4/28), respectively, which were consistent with the current clinical status of these drugs for gastric cancer therapy (Ford et al., 2014; Fujitani et al., 2016; Petrioli et al., 2016). Among these 28 cases, 27 received postoperative adjuvant chemotherapy according to the current NCCN guidelines, and the standard regimens were applied regardless of the results of zPDX assays. All these 27 patients were followed up till June 30, 2020, with 8–20 months (average 12.55 ± 2.51 months). None of these patients was dead, and 6 cases recurred during follow up. The recurrence was determined by elevated serum tumor markers or radiological examination (Table 1). We also evaluated the correlation of chemosensitive profiling in zPDXs with the clinical responses. Based on the information that any drug used in the clinical practice showed to be chemosensitive in zPDXs, these patients were divided into four groups. If at least one drug presented a good response in zPDXs (responder drug) in practical therapy, they were categorized to one, two, or three responder drug(s) groups; otherwise, they were assigned to the nonresponder drug group. As shown in Table 2, patients obtained lower recurrence if they were treated with the drug(s) with a good response in zPDXs (*P* = 0.029). For example, patient #20 was diagnosed as diffuse-type gastric adenocarcinoma with elevated preoperative serum AFP level (13.30 ng/ml) (Figures 5A,D), and his serum AFP level went down to normal range after radical surgery. He initially received postoperative oxaliplatin/paclitaxel (TP) therapy, but his serum AFP increased to 16.4 ng/ml at postoperative 6 months (Figure 5D). The image study showed one metastatic foci in the right lobe of his liver (Figure 5F), but no abnormality was found in his baseline radiological assay (Figure 5E). Thus, his tumor was considered to relapse. The regimen was adjusted to single drug capecitabine (a prodrug of 5-FU), and 5-FU was the only sensitive drug in his zPDX study (Figures 5B,C). Two cycles later, his serum AFP level returned to the normal range, and stayed for more than 5 months.

**TABLE 2:** The relationship of tumor relapse with whether the chemotherapy regimen contained responder drugs.

		No.	Tumor relapse		χ^2^	*P*
			No (21)	Yes (6)		
Chemotherapy regimen	Nonresponder drug	6	2 (33.33%)	4 (66.67%)	9.000	0.029
	One responder drug	18	16 (88.89%)	2 (11.11%)		
	Two responder drugs	2	2 (100.00%)	0 (0.00%)		
	Three responder drugs	1	1 (100.00%)	0 (0.00%)		

**Responder drug*, drug showing chemosensitivity to a certain patient’s tumor in the zPDX study.*

We further performed the risk factor analysis for early tumor relapse in these 27 patients, and we observed that responder drug(s) used or not was the only risk factor for early-stage tumor recurrence (Table 3, *P* = 0.003). All these findings with great promise collectively suggested that there was a good correlation between our zPDX assays and clinical practice.

**TABLE 3:** The risk factors for tumor relapse in the enrolled 27 patients.

Factors	No.	Tumor relapse		χ^2^	*P*
		No (21)	Yes (6)		
Age (years)				0.01	0.638
<60	13	10 (76.92%)	3 (23.08%)		
≥60	14	11 (78.57%)	3 (21.43%)		
Gender				0.220	0.502
Male	20	16 (80.00%)	4 (20.00%)		
Female	7	5 (71.43%)	2 (28.57%)		
Lauren classification				1.945	0.378
Intestinal	10	9 (90.00%)	1 (10.00%)		
Diffuse	8	5 (62.50%)	3 (37.50%)		
Mixed	9	7 (77.78%)	2 (22.22%)		
TNM stage				0.001	0.695
II	18	14 (77.78%)	4 (22.22%)		
III	9	7 (77.78%)	2 (22.22%)		
Depth of tumor invasion				0.051	0.594
T1–2	8	6 (75.00%)	2 (25.00%)		
T3–4	19	16 (84.21%)	4 (15.79%)		
Lymph node metastasis				0.622	0.406
N0	8	7 (87.50%)	1 (12.50%)		
N1–N3	19	15 (78.95%)	5 (21.05%)		
Chemotherapy regimen				8.816	0.003
≥1 responder drug	21	19 (90.48%)	2 (9.52%)		
Nonresponder drug	6	2 (33.33%)	4 (66.67%)		

**Responder drug*, drug showing chemosensitivity to a certain patient’s tumor in the zPDX study.*

## Discussion

In the preclinical study, we established a novel and ideal platform and protocol for gastric cancer drug response screening using the larvae zPDX model. We further validated that the original prominent cell components of human gastric cancer, and thus the main clinicopathological features of gastric cancer was reserved in the zPDX models in addition to our previous report (Wu et al., 2017). We reported, to our knowledge, the largest sample size of successfully established zPDXs of human gastric cancer for chemosensitivity assays. We demonstrated the feasibility of drug screening in this zPDX model and indicated for the first time that choice of appropriate sensitive drugs is the most prominent factor for postoperative early-stage relapse of gastric cancer. These promising results not only explore the prediction of chemosensitivity in zebrafish but also suggest the crucial role of zPDXs in precision cancer medicine.

As for the administration routes of drugs in zebrafish larvae, some researchers preferred microinjection (Tu et al., 2020), and the others preferred submersion in embryo medium (Brown et al., 2017; Fior et al., 2017). Zebrafish should be anesthetized for drug microinjection, but repeated anesthetization and microinjection will impair the fishes inevitably and may increase the platform instability. Thus, we investigated whether these drugs may be administrated *via* submersion. Our results showed that 5-FU, CDDP, DXT, or Dox can be administrated *via* submersion effectively. Our study approved the notion that drug delivery in larvae *via* submersion therapy makes it possible to accurately assess drug dosing, pharmacokinetics, and pharmacodynamics (Fazio et al., 2020), and this administration greatly facilitates testing of drug effects (Brown et al., 2017). However, drug penetration or accurate oral uptake remains largely unknown, and accurate drug dosing and optimized drug schedule cannot be achieved in this submersion therapy (Yan et al., 2019). We believed that our current assays are performed mainly to determine the response of xenografts to certain drugs rather than to determine their optimal doses, namely, “proof-of-concept” study (Fior et al., 2017).

Fior et al. (2017) treated zebrafish xenografts of colorectal cancer with combination therapy [FOLFIRI (5-FU+irinotecan+folinic acid) or FOLFOX (5-FU+oxaliplatin+folinic acid)] by submersion and investigated the response difference between these two regimens, while Yan et al. (2019). orally gavaged adult zebrafish xenografts of human rhabdomyosarcoma with an equivalent doses of olaparib and temozolomide. Such combination therapy in zPDXs may involve the interactions among drugs and may reflect the clinical responses of patient tumor to certain chemotherapy regimens more realistically. Our protocol evaluated the respective response to each drug for individual patient tumor and was easy to compose optimal regimen with reference to current guidelines. We will perform comparative study of the chemosensitivity results in our present protocol with the combination therapy for gastric cancer in the future.

As to evaluate the drug response in zebrafish, Fazio et al. (2020) summarized four ways, including direct imaging of unlabeled tumor cells (particularly for naturally pigmented tumors as melanoma) (Dang et al., 2016), fluorescent imaging of tumor size (Yan et al., 2019), exposing the cancer cells to viable fluorescent dyes (Fior et al., 2017), and non-invasively ultrasonography. We labeled tumor cells with viable fluorescent dyes and counted the cells before and after treatment. This approach was straightforward and convenient, but it was laborious. The development of automated injector systems and automated imaging systems for zebrafish larvae will further illustrate the high potential for automation and scaling of zPDXs, and they would be beneficial for standardization and potential clinical deployment (Tu et al., 2020).

In this preclinical study with this platform, 27 enrolled cases received postoperative chemotherapy with standard regimens according to the current guidelines, and the relapse was monitored. We analyzed the correlation of the chemosensitivity profiling in zPDXs with the patient early-stage tumor recurrence. There was lower incidence of relapse in the patients who received at least one responder drug compared with those with nonresponder drug, which indicated the nice consistency between zPDX test and clinical practice for gastric cancer. The TNM staging is generally recognized as the key factor for postoperative recurrence of gastric cancer (Smyth et al., 2020). However, our results indicated that appropriate chemotherapy regime used or not is closely associated with early-stage relapse for these specific patients with TNM II–III stage diseases. Although it is almost the common sense, our study provided the convincing evidence for this notion. Our studies validated the feasibility of our current larval zebrafish platform for drug screening for gastric cancer chemotherapy, and intensified the importance of chemotherapeutics choice for the prognosis of gastric cancer patients. Besides chemotherapy, many other anticancer agents, such as small-molecule inhibitors, have been successfully assayed in zebrafish models (Fazio et al., 2020). We also used anti-angiogenic drugs, including ramucirumab, apatinib, regorafenib, and cabozantinib, in our larval zPDXs, and showed that they exerted certain response to specific zebrafish xenografts from different patients (data not shown). Collectively, the present preclinical study indicated that our larval zPDX platform was well established, and it was practical and feasible for drug screening for gastric cancer chemotherapy.

The major advantage of PDX study is that it can reserve the characteristic tumor microenvironment. However, only a certain number of cells can be injected into zebrafish larvae. Although we and other researchers have acquired favorable results in larval zPDX studies involving several kinds of human cancers (Fior et al., 2017) and we found that CEA, CA199, and HAPLN1 are reserved in zebrafish engraftment, we are concerned about the representativeness due to restriction of the small number of human cells. A small amount cells required is thought to be an advantage of zebrafish larvae, and about 50 or more fishes are used to evaluate the response to one drug, which is deemed to be sufficient. We revealed that zPDXs retained tumor cells and fibroblasts of the original tumors; however, CD68 or MPO (myeloperoxidase) was not detected in zPDXs for the presence of myeloid cells although they were expressed in the corresponding patient tumor tissues (data not shown). Moreover, engrafted zebrafish are kept at nonphysiological temperatures of ≤34°C (Yan et al., 2019) and thus the cancer cells do not proliferate at similar rates as when grown in mouse or human. Most of chemotherapeutics work at physiological temperature. The influence of temperature on drug sensitivity remains unclear. The engraftment studies are necessarily confined to studies before 10 dpf because fish eventually develop acquired immune responses that kill human cancer cells. Recently, Yan et al. (2019) have developed an optically clear, immune-deficient *prkdc^–/–^, il2rga^–/–^* zebrafish, and their immune-deficiency persists for more than 28 dpf. The adult zebrafish are reared at 37°C and can be engrafted about 5 × 10^4^ cells per fish. This new zebrafish opens new avenues for personalized therapy.

We report herein the promising drug screening study in zebrafish models for gastric cancer with the largest sample size to date. Just as the first ongoing zPDX-related clinical trial (NCT03668418)^2^, the prospective clinical trials are needed to validate the predictability of larval zPDXs for gastric cancer chemotherapy. Given the current encouraging results in zPDX studies and the advance in approaches, the distinctive role of zebrafish in precision cancer medicine may be anticipated, and it is possible that, 1 day, a fish could help to save our lives (Xiao et al., 2020).

## Conclusion

In the present study, we developed a stable and reliable protocol using zPDXs to screen individualized chemotherapeutics for gastric cancer patients. The chemosensitivity obtained from zPDXs was well consistent with the clinical responses, and the responder drug(s) from zPDXs used or not accounted for the unique risk factor for postoperative early-stage recurrence in these patients. Our translational findings strongly support the idea that zPDXs help to predict the chemotherapeutics response and to achieve precise chemotherapy for gastric cancer.

## Data Availability Statement

The original contributions presented in the study are included in the article/Supplementary Material, further inquiries can be directed to the corresponding author/s.

## Ethics Statement

The studies involving human participants were reviewed and approved by the Institutional Review Board of Nanjing University of Chinese Medicine. The patients/participants provided their written informed consent to participate in this study. The animal study was reviewed and approved by the Institutional Animal Care and Use Committee (IACUC) of Nanjing University of Chinese Medicine.

## Author Contributions

LS, JZ, and MH conceptualized the study. JZ, JW, YW, AT, and XY developed the methodology. JZ, JW, YW, RF, GX, FW, YH, and SQ performed the experiments. LS, JZ, and MH analyzed the data. LS and JZ wrote the manuscript. All authors contributed to the article and approved the submitted version.

## Conflict of Interest

AT was employed by the company Nanjing Amory Biotech Co. The remaining authors declare that the research was conducted in the absence of any commercial or financial relationships that could be construed as a potential conflict of interest.

## Footnotes

1 https://www.drugbank.ca/drugs

2 https://clinicaltrials.gov/ct2/show/NCT03668418(2018)

## Supplementary Material

Supplementary Figure 1 Schematic diagram of establishment of zPDXs and preclinical personalized drug screening.

Supplementary Figure 2 Treatment of 5-FU, CDDP, DXT, or Dox inhibited tumor proliferation in zPDXs of certain patients. Treatment of 5-FU, CDDP, DXT, or Dox exerted the significant inhibition of tumor proliferation in zPDXs of patient #6, #15, #56, and #29, respectively (^∗∗^*P* < 0.01, ^∗∗∗^*P* < 0.001) (Engrafted tumor cells were dyed with red fluoresce using DiL).
