# Supplementary material for: Prediction of Sensitivity and Efficacy of Clinical Chemotherapy Using Larval Zebrafish Patient-Derived Xenografts of Gastric Cancer
Source: Front Cell Dev Biol. 2021 Jun 7;9:680491. doi: 10.3389/fcell.2021.680491 (PMC8215369; doi:10.3389/fcell.2021.680491)
Supplement: Supplementary file 1 [file Table_1.DOCX]

**Table S1. The detailed clinicopathological features of gastric cancer patients enrolled in this study**

| Patient No. | Age (y) | Gander | Preoperative serum tumor markers | | | Gastrectomy Type ^1^ | pTNM  stage | T  stage | N  stage | Lauren  classification | Her-2 ^2^ | Postoperative serum tumor markers ^3^ | | |
| --- | --- | --- | --- | --- | --- | --- | --- | --- | --- | --- | --- | --- | --- | --- |
|  |  |  | CEA (ng/ml) | AFP (ng/ml) | CA199 (U/ml) |  |  |  |  |  |  | CEA (ng/ml) | AFP (ng/ml) | CA199 (U/ml) |
| #1 | 66 | male | 3.07 | 2.90 | 8.39 | Total | IIB | 3 | 1(2/40) | Mixed | 0 | 3.43 | 2.4 | 3.68 |
| #2 | 54 | male | 1.24 | 2.40 | 3.62 | Distal | IA | 1b | 0(0/16) | Intestinal | 1+ | 1.2 | 2.21 | 3.76 |
| #3 | 66 | female | 1.00 | 3.9 | 8.57 | Distal | IIIB | 3 | 3a(14/41) | Mixed | 2+ | 1.00 | 4.2 | 2.65 |
| #4 | 71 | male | 1.00 | 3.20 | 2.14 | Total | IIA | 3 | 0(0/44) | Intestinal | 2+ | 1.00 | 3.5 | 3.37 |
| #5 | 44 | female | 1.00 | 2.10 | 3.19 | Total | IIIC | 3 | 3b(25/29） | Intestinal | 1+ | 1.00 | 2.30 | 3.00 |
| #6 | 83 | male | 1.46 | 1.60 | 8.02 | Total | IIA | 3 | 0(0/35) | Intestinal | 3+ | 1.23 | 1.23 | 7.42 |
| #7 | 72 | female | 1.08 | 1.40 | 142.65 | Distal | IIIA | 3 | 3a(7/42) | Diffuse | 0 | 2.67 | 2.67 | 21.54 |
| #8 | 79 | female | 2.54 | 3.21 | 23.67 | Distal | IIA | 3 | 0(0/60) | Intestinal | 1+ | 3.23 | 4.23 | 12.89 |
| #9 | 74 | male | 1.43 | 2.50 | 2.00 | Total | IIB | 3 | 1(1/35) | Intestinal | 1+ | 1.56 | 2.76 | 2.00 |
| #10 | 54 | female | 1.00 | 2.30 | 5.41 | Total | IIIB | 3 | 3a(13/43) | Diffuse | 2+ | 1.00 | 2.00 | 2.00 |
| #11 | 39 | female | 1.00 | 1.60 | 5.44 | Distal | IIIA | 3 | 2(5/40) | Mixed | 0 | 1.24 | 2.70 | 5.76 |
| #12 | 71 | female | 1.42 | 2.30 | 4.06 | Distal | IIIA | 3 | 2(4/41) | Diffuse | 1+ | 2.30 | 2.51 | 6.78 |
| #13 | 59 | female | 1.35 | 5.20 | 2.00 | Distal | IIIC | 3 | 3b(26/31) | Diffuse | 1+ | 1.10 | 4.10 | 3.39 |
| #14 | 78 | female | 2.70 | 3.60 | 2.00 | Total | IIA | 3 | 0(0/29) | Diffuse | 0 | 3.35 | 2.10 | 3.12 |
| #15 | 56 | male | 4.36 | 2.40 | 46.56 | Total | IIIC | 4a | 3b(67/91) | Diffuse | 1+ | 3.27 | 2.45 | 12.33 |
| #16 | 67 | male | 1.43 | 2.40 | 5.27 | Total | IIA | 3 | 0(0/27) | Diffuse | 2+ | 2.03 | 2.87 | 6.12 |
| #17 | 56 | male | 1.00 | 4.30 | 2.00 | Distal | IIA | 3 | 0(0/53) | Intestinal | 0 | 1.19 | 2.60 | 2.73 |
| #18 | 65 | male | 34.88 | 4.00 | 560.61 | Total | III | 3 | 0(5/48) | Diffuse | 2+ | 7.97 | 3.30 | 139.80 |
| #19 | 61 | male | 1.49 | 5.20 | 18.57 | Total | IIIB | 4a | 3a(8/19) | Diffuse | 1+ | 1.22 | 3.60 | 9.01 |
| #20 | 56 | male | 2.53 | 13.30 | 5.71 | Distal | IIB | 3 | 1(1/50) | Diffuse | 0 | 1.51 | 3.30 | 2.44 |
| #21 | 71 | male | 2.46 | 2.20 | 2.00 | Total | IIB | 3 | 1(1/36) | Diffuse | 0 | 1.96 | 3.51 | 2.35 |
| #22 | 64 | male | 1.50 | 2.00 | 2.00 | Total | IIA | 3 | 0(0/37) | Intestinal | 0 | 1.00 | 1.80 | 2.20 |
| #23 | 50 | male | 2.49 | 2.30 | 9.88 | Total | IIB | 2 | 0(0/34) | Intestinal | 2+ | 2.2 | 2.19 | 7.13 |
| #24 | 76 | female | 1.00 | 2.10 | 2.00 | Total | IIA | 3 | 0(0/39) | Intestinal | 1+ | 1 | 1.90 | 2.00 |
| #25 | 75 | male | 3.01 | 1.90 | 6.01 | Distal | IIB | 3 | 1(2/23) | Diffuse | 1+ | 2.55 | 1.80 | 4.31 |
| #26 | 59 | male | 1.65 | 4.20 | 15.35 | Total | IB | 2 | 0(0/61) | Mixed | 1+ | 1.23 | 2.90 | 14.15 |
| #27 | 54 | male | 1.47 | 6.00 | 5.23 | Total | IIB | 3 | 1(1/43) | Diffuse | 0 | 1.66 | 3.20 | 5.06 |
| #28 | 58 | male | 1.83 | 2.10 | 3.69 | Total | IIIC | 4a | 3b(25/39) | Mixed | 0 | 1.83 | 2.23 | 3.12 |
| #29 | 74 | female | 1.11 | 1.30 | 2.00 | Total | IIIA | 3 | 2(5/68) | Intestinal | 3+ | 1.64 | 1.50 | 2.00 |
| #30 | 78 | male | 2.72 | 3.20 | 15.83 | Total | IIIC | 4a | 3b(36/71) | Mixed | 2+ | 3.03 | 2.50 | 18.37 |
| #31 | 53 | male | 1.46 | 4.70 | 125.08 | Total | III | 3 | 1(1/61) | Mixed | 2+ | 1.12 | 4.90 | 2.00 |
| #32 | 65 | male | 9.30 | 4.00 | 91.67 | Total | IIB | 2 | 2(5/58) | Intestinal | 3+ | 4.25 | 6.40 | 23.30 |
| #33 | 73 | male | 12.91 | 1.60 | 6.26 | Total | IIIA | 3 | 2(6/32) | Mixed | 0 | 10.6 | 4.25 | 5.12 |
| #34 | 67 | male | 2.80 | 3.00 | 9.32 | Total | IIA | 3 | 0(0/46) | Mixed | 0 | 2.00 | 3.00 | 0.76 |
| #35 | 48 | female | 3.27 | 3.70 | 9.09 | Total | IIIB | 1a | 3b(28/49) | Intestinal | 0 | 3.22 | 3.00 | 7.48 |
| #36 | 59 | male | 3.35 | 1.40 | 15.25 | Distal | IB | 2 | 0(0/25) | Intestinal | 0 | 2.21 | 1.20 | 8.71 |
| #37 | 82 | male | 2.41 | 354.00 | 2.31 | Total | IIIB | 3 | 3a(7/44) | Mixed | 1+ | 2.89 | 4.89 | 3.45 |
| #38 | 57 | male | 4.26 | 6.70 | 2.91 | Total | IIIB | 3 | 3a(13/30) | Mixed | 1+ | 1.96 | 6.60 | 3.93 |
| #39 | 54 | male | 1.54 | 3.30 | 6.22 | Total | IB | 1 | 1(2/38) | Intestinal | 1+ | 1.00 | 3.30 | 4.33 |
| #40 | 81 | male | 1.39 | 2.00 | 3.72 | Total | IIIA | 3 | 2(5/40) | Mixed | 0 | 3.88 | 2.80 | 5.99 |
| #41 | 56 | female | 9.00 | 2.70 | 8.23 | Total | IIIB | 3 | 3(10/18) | Mixed | 2+ | 1.00 | 1.90 | 3.41 |
| #42 | 75 | male | 2.01 | 1.90 | 31.09 | Total | IIIB | 3 | 2(3/37) | Intestinal | 1+ | 3.56 | 4.6 | 124.77 |
| #43 | 63 | male | 44.86 | 3.20 | 2.00 | Total | IIB | 2 | 2(5/47) | Mixed | 0 | 3.00 | 1.30 | 2.00 |
| #44 | 55 | male | 2.50 | 10.10 | 2.00 | Total | IIIC | 3 | 3b(19/37) | Mixed | 2+ | 4.19 | 7.60 | 2.00 |
| #45 | 56 | female | 1.09 | 1.90 | 33.00 | Distal | IIA | 3 | 0(0/46) | Mixed | 0 | 2.12 | 2.10 | 15.12 |
| #46 | 70 | male | 3.36 | 4.80 | 11.66 | Total | III | 3 | 3(7/27) | Diffuse | 2+ | 3.43 | 2.42 | 21.34 |
| #47 | 75 | male | 1.80 | 1.90 | 2.00 | Total | IIIC | 4 | 3b(20/35) | Diffuse | 1+ | 2.33 | 1.90 | 21.30 |
| #48 | 62 | male | 3.60 | 114.00 | 2.00 | Total | IIB | 2 | 2(5/30) | Diffuse | 3+ | 2.09 | 31.5 | 2.00 |
| #49 | 57 | male | 1.00 | 5.80 | 4.30 | Distal | III | 4a | 3b(20/49) | Diffuse | 0 | 1.00 | 6.50 | 5.71 |
| #50 | 54 | male | 3.07 | 5.90 | 3.39 | Total | III | 4a | 1(4/62) | Mixed | 0 | 1.53 | 5.70 | 4.13 |
| #51 | 73 | male | 5.24 | 2.90 | 20.24 | Total | IIIA | 4a | 2(4/34) | Mixed | 2+ | 1.40 | 3.00 | 4.40 |
| #52 | 56 | male | 2.09 | 2.40 | 18.78 | Distal | IIA | 3 | 0(0/24) | Diffuse | 0 | 1.35 | 1.60 | 13.00 |
| #53 | 76 | male | 2.60 | 3.50 | 3.77 | Total | IIA | 2 | 1(1/43) | Mixed | 1+ | 2.83 | 3.22 | 2.19 |
| #54 | 82 | male | 1.50 | 2.40 | 4.94 | Distal | IIIB | 3 | 3a (7/36) | Intestinal | 1+ | 2.35 | 2.11 | 3.12 |
| #55 | 68 | female | 8.40 | 1.70 | 971.38 | Distal | IIA | 3 | 0(0/16) | Mixed | 1+ | 1.00 | 1.90 | 15.55 |
| #56 | 81 | female | 13.26 | 3.10 | 197.82 | Total | IIIA | 3 | 2(3/45) | Intestinal | 1+ | 4.27 | 3.6 | 3.79 |

^1^ All these patients have undergone radical gastrectomy for cure intent. The surgical type mainly depended on the tumor location. “Total” means total gastrectomy, and “Distal” means subtotal distal gastrectomy.

^2^ Her-2 expression in primary tumor tissues was determined by IHC. (“0”means no Her-2 was detected, and “1+~3+” means Her-2 was detected at different levels.)

^3^ Postoperative serum tumor markers were measured on 4 weeks after operation, which was prior to postoperative adjuvant chemotherapy.
