# Supplementary material for: Prediction of Sensitivity and Efficacy of Clinical Chemotherapy Using Larval Zebrafish Patient-Derived Xenografts of Gastric Cancer
Source: Front Cell Dev Biol. 2021 Jun 7;9:680491. doi: 10.3389/fcell.2021.680491 (PMC8215369; doi:10.3389/fcell.2021.680491)
Supplement: Supplementary file 2 [file Table_2.DOCX]

**Table S2. The expression statuses of CEA, CA199 and HAPLN1 in zPDXs of gastric cancer**

| Patient No. | CEA | | CA199 | | HAPLN1 | |
| --- | --- | --- | --- | --- | --- | --- |
|  | patient tumor | zPDX | patient tumor | zPDX | patient tumor | zPDX |
| #15 |  |  | **+** | **+** | **+** | **+** |
| #32 |  |  | **+** | **+** | **+** | **+/-** |
| #41 | **+** | **+** |  |  | **+** | **+** |
| #43 | **+** | **+** |  |  | **+** | **+/-** |
| #55 |  |  | **+** | **+** | **+** | **+** |
| #56 | **+** | **+** | **+** | **+** | **+** | **+** |
