# Supplementary material for: Prediction of Sensitivity and Efficacy of Clinical Chemotherapy Using Larval Zebrafish Patient-Derived Xenografts of Gastric Cancer
Source: Front Cell Dev Biol. 2021 Jun 7;9:680491. doi: 10.3389/fcell.2021.680491 (PMC8215369; doi:10.3389/fcell.2021.680491)
Supplement: Supplementary file 3 [file Table_3.DOCX]

**Table S3. The detailed results of zPDX of gastric cancer patients enrolled in this study**

| Patient No. | Tumor recurrence ^1^ | | | Postoperative adjuvant chemotherapy ^2^ | | Follow-up  (m) | zPDX ^3^ | | |
| --- | --- | --- | --- | --- | --- | --- | --- | --- | --- |
|  | Status | Time (m) | Evidence | Regimen | Regimen changing |  | Status | Reason of failure | Chemosensitivity results |
| #1 | NO |  |  | NO | NO | 20 | success |  | All resistance |
| #2 | NO |  |  | NO | NO | 18 | fail | 1 |  |
| #3 | NO |  |  | FOLFORI | NO | 15 | fail | 2 |  |
| #4 | NO |  |  | NO | NO | 15 | fail | 1 |  |
| #5 | NO |  |  | FLOT( only 3 cycles) | NO | 15 | fail | 1 |  |
| #6 | NO |  |  | Capecitabine | NO | 18 | success |  | CDDP^***^, 5-FU^**^, Dox^*^, DXT^r^ |
| #7 | NO |  |  | CAPEOX | NO | 15 | fail | 3 |  |
| #8 | NO |  |  | Capecitabine | NO | 14 | fail | 1 |  |
| #9 | NO |  |  | CAPEOX | NO | 17 | success |  | 5-FU^*^, CDDP^*^, DXT^*^, Dox^r^ |
| #10 | NO |  |  | FLOT | NO | 14 | fail | 1 |  |
| #11 | NO |  |  | FOLFORI | NO | 15 | success |  | DXT^***^, 5-FU^*^, CDDP^r^, Dox^r^ |
| #12 | NO |  |  | CAPEOX | NO | 13 | fail | 1 |  |
| #13 | NO |  |  | CAPEOX | NO | 13 | fail | 4 |  |
| #14 | YES | 10 (die) | CT (abdomen) | NO | NO | 13 | fail | 1 |  |
| #15 | NO |  |  | CAPEOX | NO | 15 | success |  | 5-FU^***^, DXT^***^, CDDP^r^, Dox^r^ |
| #16 | NO |  |  | Capecitabine | NO | 14 | success |  | 5-FU^***^, DXT^***^, CDDP^r^, Dox^r^ |
| #17 | NO |  |  | TP | NO | 14 | fail | 1 |  |
| #18 | YES | 5 | CEA↑(10.23) | TP | FLOT | 14 | fail | 1 |  |
| #19 | YES | 6 | MR (lymph nodes) | FLOT | Irinotecan | 14 | fail | 2 |  |
| #20 | YES | 6 | AFP↑(16.40) | TP | Capecitabine | 14 | success |  | 5-FU^*^, DXT^r^, CDDP^r^, Dox^r^ |
| #21 | NO |  |  | NO | NO |  | fail | 1 |  |
| #22 | NO |  |  | TP | NO | 13 | fail | 4 |  |
| #23 | NO |  |  | Capecitabine | NO | 14 | success |  | All resistance |
| #24 | NO |  |  | Capecitabine | NO | 12 | fail | 4 |  |
| #25 | NO |  |  | FOLFORI | NO | 14 | success |  | 5-FU^*^, DXT^r^, CDDP^r^, Dox^r^ |
| #26 | NO |  |  | Capecitabine | NO | 12 | fail | 1 |  |
| #27 | YES | 6 | CEA↑(5.40) | FOLFORI | FOLFOX | 14 | success |  | CDDP^***^, 5-FU^***^, DXT^***^, Dox^***^ |
| #28 | YES | 6 | CEA↑(6.46) | TP | CAPEOX | 12 | fail | 1 |  |
| #29 | NO |  |  | Capecitabine | NO | 13 | success |  | 5-FU^***^, CDDP^***^, Dox^***^, DTX^***^ |
| #30 | YES | 6 | CEA↑(6.15) | NO | Capecitabine | 12 | fail | 4 |  |
| #31 | NO |  |  | CAPEOX | NO | 12 | fail | 2 |  |
| #32 | NO |  |  | FLOT | NO | 13 | success |  | DXT^***^, 5-FU^***^, CDDP^r^, Dox^r^ |
| #33 | YES | 6 | CEA↑(15.80) | Capecitabine | NO | 11 | fail | 2 |  |
| #34 | NO |  |  | Capecitabine | NO | 12 | success |  | 5-FU^*^, Dox^r^, CDDP^r^, DXT^r^ |
| #35 | YES | 12 | CEA↑(6.25) | FLOT | TP | 12 | success |  | 5-FU^*^, DXT^r^, Dox^r^, CDDP^r^ |
| #36 | NO |  |  | FOLFORI | NO | 12 | success |  | DXT^*^, CDDP^*^, Dox^r^, 5-FU^r^ |
| #37 | NO |  |  | Capecitabine | NO | 11 | fail | 2 |  |
| #38 | NO |  |  | DCF | NO | 12 | success |  | 5-FU^*^, DXT^r^, Dox^r^, CDDP^r^ |
| #39 | NO |  |  | FLOT | NO | 11 | success |  | 5-FU^*^, DTX^*^, CDDP^*^, Dox^r^ |
| #40 | NO |  |  | Capecitabine | NO | 12 | fail | 3 |  |
| #41 | NO |  |  | FOLFORI | NO | 11 | success |  | 5-FU^*^, DXT^*^, CDDP^r^, Dox^r^ |
| #42 | NO |  |  | NO | NO | 11 | fail | 4 |  |
| #43 | YES | 7 | CT (lymph nodes) | FLOT | NO | 11 | success |  | All resistence |
| #44 | YES | 7 | CT (abdomen)  Die at 9m. | FLOT | FOLFORI | 10 | fail | 4 |  |
| #45 | NO |  |  | FOLFOX | NO | 11 | success |  | CDDP^*^, DXT^*^, Dox^*^, 5-FU^r^ |
| #46 | YES | 9 | CEA↑(11.64)  CT (lymph nodes) | CAPEOX | FOLFORI | 10 | fail | 2 |  |
| #47 | YES | 7 | CEA↑(10.64)  Die at 9m. | Capecitabine | NO | 10 | success |  | All resistence |
| #48 | NO |  |  | FOLFOX | NO | 10 | success |  | 5-FU^*^, DXT^r^, Dox^r^, CDDP^r^ |
| #49 | NO |  |  | FLOT | NO | 10 | fail | 2 |  |
| #50 | NO |  |  | FLOT | NO | 10 | success |  | 5-FU^**^, Dox^r^, CDDP^r^, DXT^r^ |
| #51 | NO |  |  | TP | NO | 10 | fail | 2 |  |
| #52 | NO |  |  | TP | NO | 10 | success |  | DXT^*^, CDDP^r^, 5-FU^r^, Dox^r^ |
| #53 | NO |  |  | Capecitabine | NO | 8 | success |  | 5-FU^*^, CDDP^*^, Dox^r^, DXT^r^ |
| #54 | NO |  |  | Capecitabine | NO | 8 | success |  | CDDP^***^, DXT^***^, 5-FU^***^, Dox^r^ |
| #55 | YES | 5 | CT (lymph nodes) | FOLFOX | FLOT | 8 | success |  | All resistence |
| #56 | NO |  |  | Capecitabine | NO | 8 | success |  | DXT^**^, 5-FU^**^, CDDP^r^, Dox^r^ |

^1^ Tumor recurrence was monitored continuously by measuring serum tumor markers or image study. “YES” means recurrence, and we showed the recurrence time (months after surgery) and the evidences.

^2^ Most patients have undergone postoperative adjuvant chemotherapy, and some patients’ regimens were changed due to recurrence or other reasons. (“NO” means the patient did not receive adjuvant chemotherapy or the patient’s regimen did not change during therapy.)

^3^ In this study, the zPDX models were established in 28 cases successfully. We showed the reasons for any failure to model establishment (“1”, “2”, “3”, and “4” means “sample pollution”, “insufficient cell viability”, “needle blocking”, “unskilled technician” respectively.) . In the 28 cases, the chemosensitivity of 5-FU (5-fluorouracil), CDDP (cisplatin), DXT (docetaxel) and Dox (doxorubicin) was obtained (“*~***” means the treatment inhibited cell proliferation significantly, *: *P*<0.05, **: *P*<0.01 and ***: *P*<0.001. “r” means resistance to the drug. “All resistance” means resistance to these four drugs.).
