# Supplementary figures and images for: Prediction of Sensitivity and Efficacy of Clinical Chemotherapy Using Larval Zebrafish Patient-Derived Xenografts of Gastric Cancer
Source: Front Cell Dev Biol. 2021 Jun 7;9:680491. doi: 10.3389/fcell.2021.680491 (PMC8215369; doi:10.3389/fcell.2021.680491)

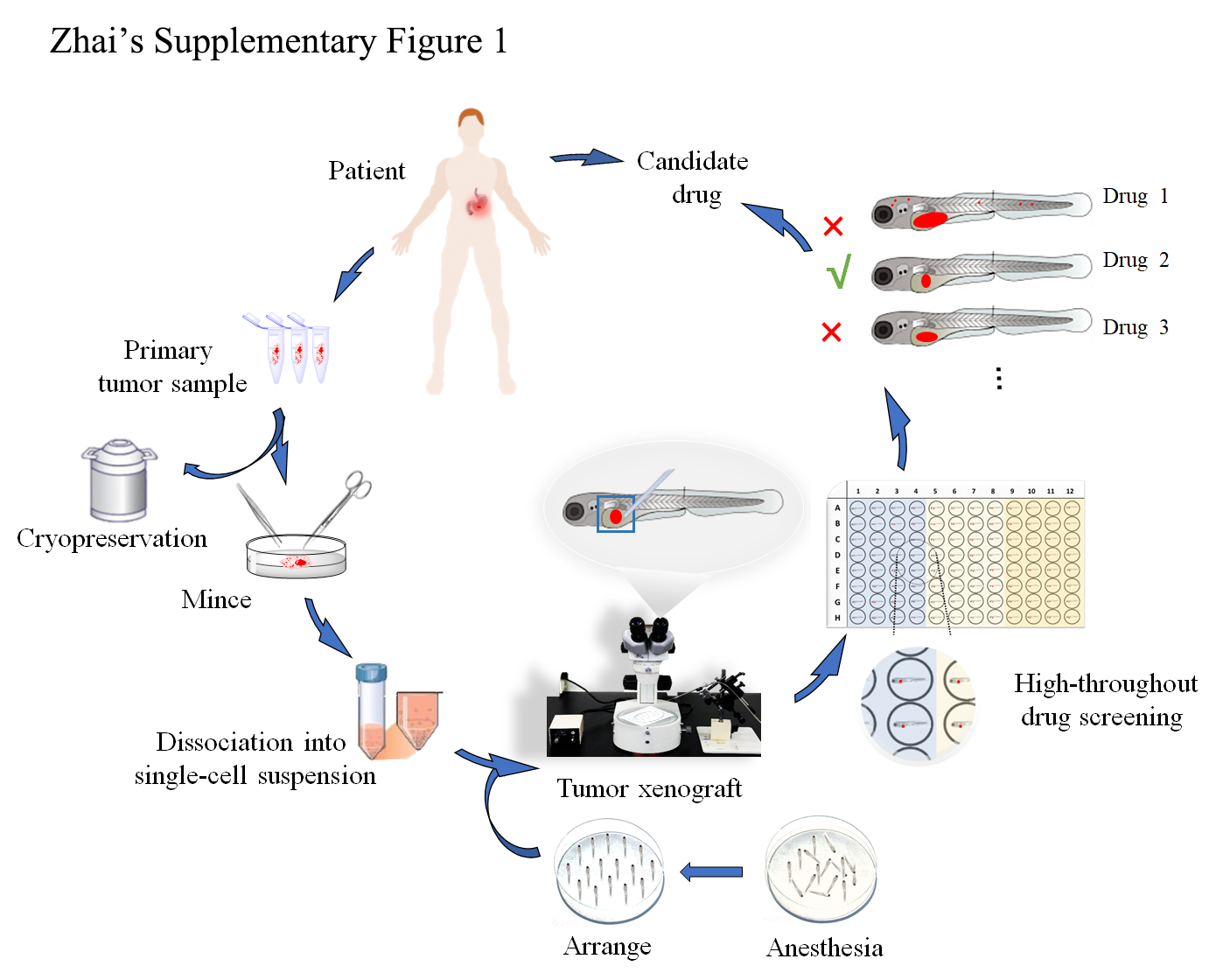

Supplement: Supplementary Figure 1 — Schematic diagram of establishment of zPDXs and preclinical personalized drug screening. [file Image_1.TIF]

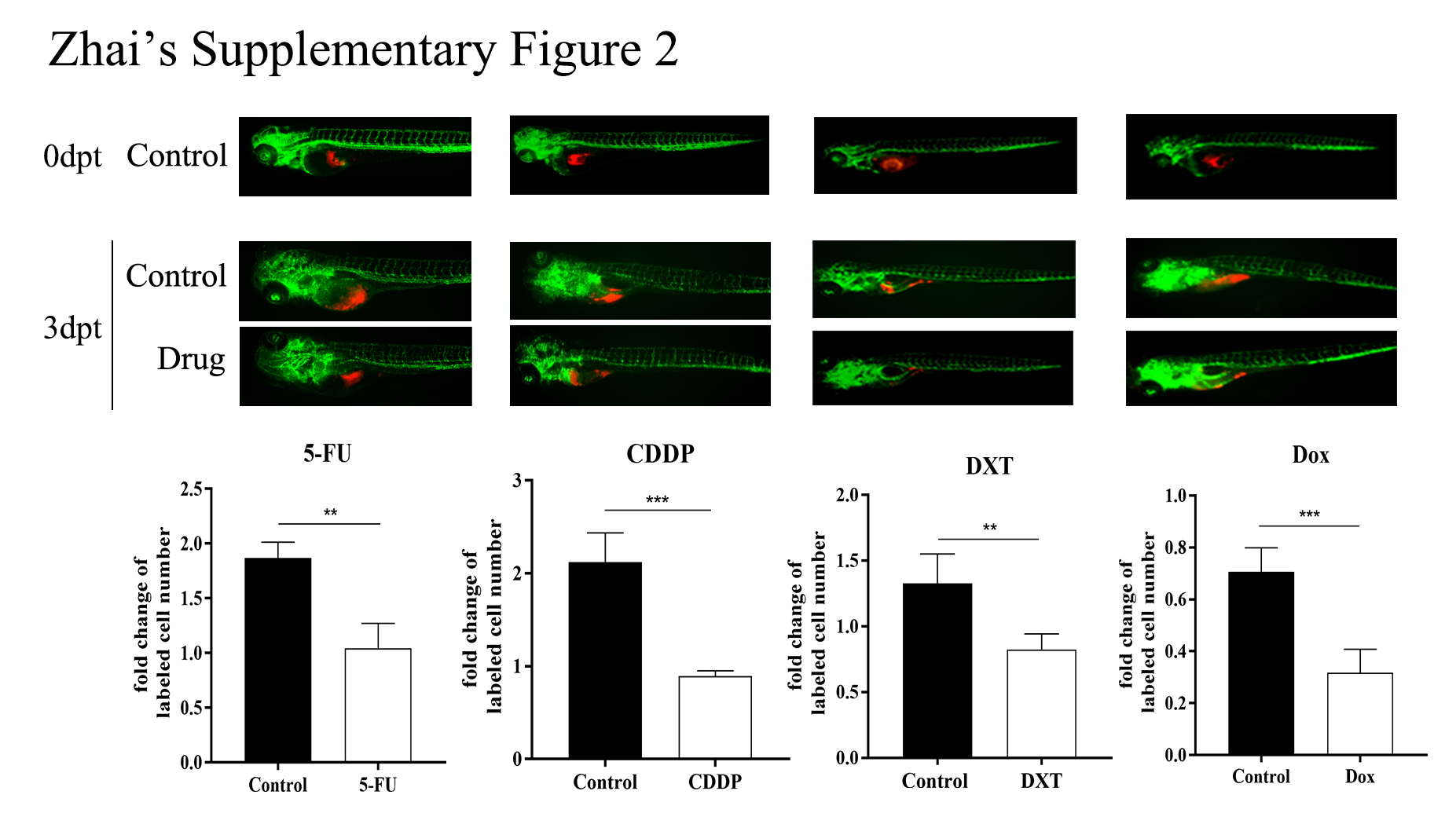

Supplement: Supplementary Figure 2 — Treatment of 5-FU, CDDP, DXT, or Dox inhibited tumor proliferation in zPDXs of certain patients. Treatment of 5-FU, CDDP, DXT, or Dox exerted the significant inhibition of tumor proliferation in zPDXs of patient #6, #15, #56, and #29, respectively (∗∗P < 0.01, ∗∗∗P < 0.001) (Engrafted tumor cells were dyed with red fluoresce using DiL). [file Image_2.TIF]
